# Testing Evolutionary and Dispersion Scenarios for the Settlement of the New World

**DOI:** 10.1371/journal.pone.0011105

**Published:** 2010-06-14

**Authors:** Mark Hubbe, Walter A. Neves, Katerina Harvati

**Affiliations:** 1 Instituto de Investigaciones Arqueológicas y Museo, Universidad Católica del Norte, San Pedro de Atacama, Chile; 2 Laboratório de Estudos Evolutivos Humanos, Departamento de Genética e Biologia Evolutiva, Instituto de Biociências, Universidade de São Paulo, São Paulo, Brazil; 3 Paleoanthropology Section, Institut für Uhr- und Frühgeschichte und Archaeologie des Mittelalters and Senckenberg Center for Human Evolution and Paleoecology, University of Tübingen, Tübingen, Germany; University of Utah, United States of America

## Abstract

**Background:**

Discussion surrounding the settlement of the New World has recently gained momentum with advances in molecular biology, archaeology and bioanthropology. Recent evidence from these diverse fields is found to support different colonization scenarios. The currently available genetic evidence suggests a “single migration” model, in which both early and later Native American groups derive from one expansion event into the continent. In contrast, the pronounced anatomical differences between early and late Native American populations have led others to propose more complex scenarios, involving separate colonization events of the New World and a distinct origin for these groups.

**Methodology/Principal Findings:**

Using large samples of Early American crania, we: 1) calculated the rate of morphological differentiation between Early and Late American samples under three different time divergence assumptions, and compared our findings to the predicted morphological differentiation under neutral conditions in each case; and 2) further tested three dispersal scenarios for the colonization of the New World by comparing the morphological distances among early and late Amerindians, East Asians, Australo-Melanesians and early modern humans from Asia to geographical distances associated with each dispersion model. Results indicate that the assumption of a last shared common ancestor outside the continent better explains the observed morphological differences between early and late American groups. This result is corroborated by our finding that a model comprising two Asian waves of migration coming through Bering into the Americas fits the cranial anatomical evidence best, especially when the effects of diversifying selection to climate are taken into account.

**Conclusions:**

We conclude that the morphological diversity documented through time in the New World is best accounted for by a model postulating two waves of human expansion into the continent originating in East Asia and entering through Beringia.

## Introduction

It has been repeatedly observed that Early American (hereafter Paleoamerican) cranial morphology differs significantly from that of recent Native Americans (hereafter called Amerindians) [1]–[3]. While Paleoamericans show strong morphological affinities with Australo-Melanesians, Amerindians are clearly associated with East Asians. These marked cranial differences have led some researchers to develop a scenario of distinct origins for early and later American groups, and to propose two waves of population expansion into the New World [2]–[4]. This view is at odds with most of the genetic evidence currently available on Native American origins, the majority of which [5]–[7] - though not all [8] - suggest a single origin for New World populations. Under this interpretation the large anatomical differences between Paleoamericans and Amerindians stem either from *in situ* stochastic microevolutionary processes [9], or from progressive loss of the diversity originally present in the mother-population at the end of the Pleistocene [10], rather than from distinct ancestry of the two groups.

Since cranial morphology has been shown to reflect population history in recent human populations [11]–[17], it is suitable for assessing dispersion patterns for prehistoric groups for which molecular information is not available. Our study: a) demonstrates that Paleoamericans and Amerindians exhibit distinct morphological patterns; and b) tests different colonization scenarios by formulating predictions of morphological differentiation associated with different temporal and geographic constraints under different evolutionary and dispersion models into the New World.

**Figure 1:**
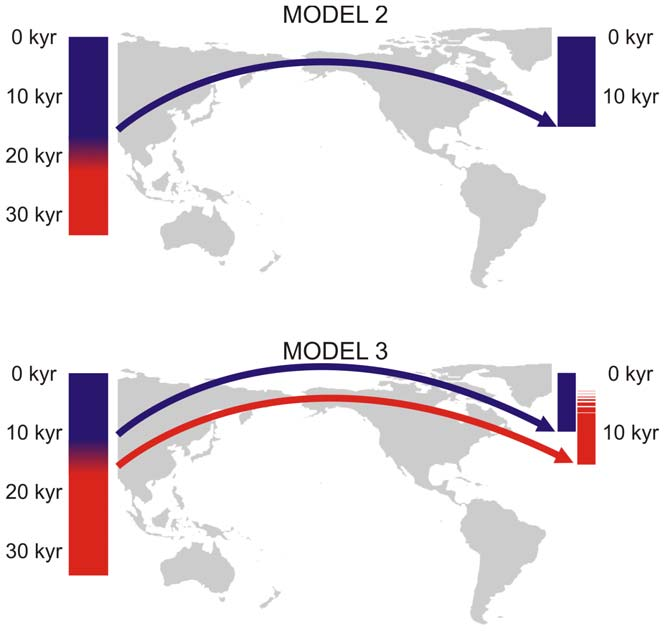
Representation of the geographic dispersion models tested for the occupation of the Americas. Model 1 is not represented because it is a control model (assuming direct linear distances among all groups). The bars represent the morphological change observed in East Asia (left) and America (right) during Late Pleistocene/Early Holocene. The red color represents the morphology present in Asia by the end of the Pleistocene and the blue color represents the morphology present nowadays in Asia and America. Model 2 assumes that the morphological differentiation in East Asia occurred before America's settlement and that the New World was occupied only once; Model 3 assumes two distinct dispersions into the continent. See text for detailed description of each model. The dates presented are just approximations, but they assume America's settlement to have occurred around 15 kyr BP (19–22).

Our approach is twofold: First we calculated the predicted rate of morphological evolution [18] under three scenarios: 1) Paleoamericans are the direct ancestors of Amerindians; 2) Paleoamericans and Amerindians share a last common ancestor by the time of the first entrance into the continent (∼15 kyr BP [19]–[22]); and 3) the last common ancestor between them was East Asian and predated the first dispersal into the continent (represented by the Late Pleistocene Zhoukoudian Upper Cave individuals; ∼30–11 kyr BP [23]–[27]). Secondly, we compared the D^2^ matrix among a large number of modern human populations to the geographic distance matrices among the same populations as predicted under three dispersion models (Figure 1). We did this in order to evaluate which model best explains the observed morphological differences between Paleoamericans and Amerindians, taking into account the likely ancestral morphology for the region (Upper Cave [23]–[27]). Model 1 is the control and comprises the direct linear geographic distances between our population samples. Model 2 assumes a single dispersal event into the New World; this model considers Paleoamericans as direct local ancestors to Amerindians. Model 3 assumes that Paleoamericans and Amerindians share an ancestor outside the New World. Following the archaeological consensus that the route of entrance to the Americas was through the Bering Strait [4], [24]–[27] this model assumes that both morphological patterns originated from two distinct migration waves from Northeast Asia.

In order to account for the effects of selection to climate in generating the morphological differences observed, analyses were ran twice: once using a set of 24 cranial variables; and a second time excluding five variables previously shown to respond to diversifying selection to climate [13], [17] and thus subject to higher rates of homoplastic similarities among human groups living in similar environments. If the cranial differences observed between Paleoamericans and Amerindians are primarily due to climatic selective pressures, one would expect that removing these variables would decrease their differences and strengthen the models representing a single colonization event of the continent (Model 2).

## Results

**Figure 2:**
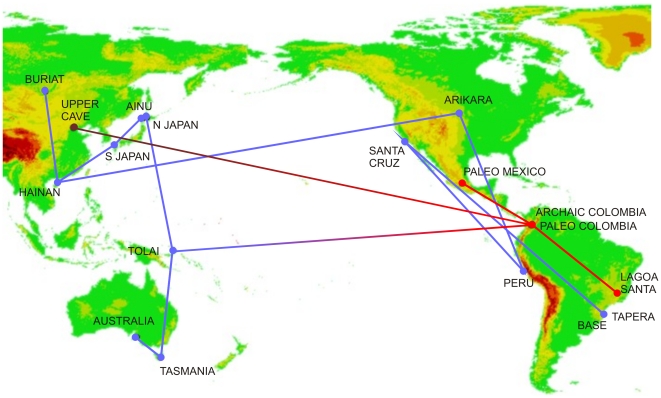
Minimum Spanning Tree of the series calculated from the Mahalanobis squared distances among groups and plotted over their geographic coordinates. The lines represent the closest path for connecting all samples according to the morphological distances between them. Red dots represent samples with Paleoamerican morphology and the brown dot represents the specimens from Zhoukoudian Upper Cave. Blue dots indicate the Late Holocene samples from East Asia, the Americas and Australo-Melanesia.

Figure 2 shows the geographic location of the population samples and the nearest neighbor connections based on Mahalanobis D^2^ (Table S1). Only the connections based on 24 variables are shown, since both analyses had very similar results. Paleoamericans and Amerindians clearly show distinct morphologies: none of the former samples are directly connected to the latter. Paleoamericans are instead linked by nearest morphological distance to the early modern humans from Zhoukoudian Upper Cave and to Australo-Melanesians, while Amerindians are joined with East Asian groups.

**Figure 3:**
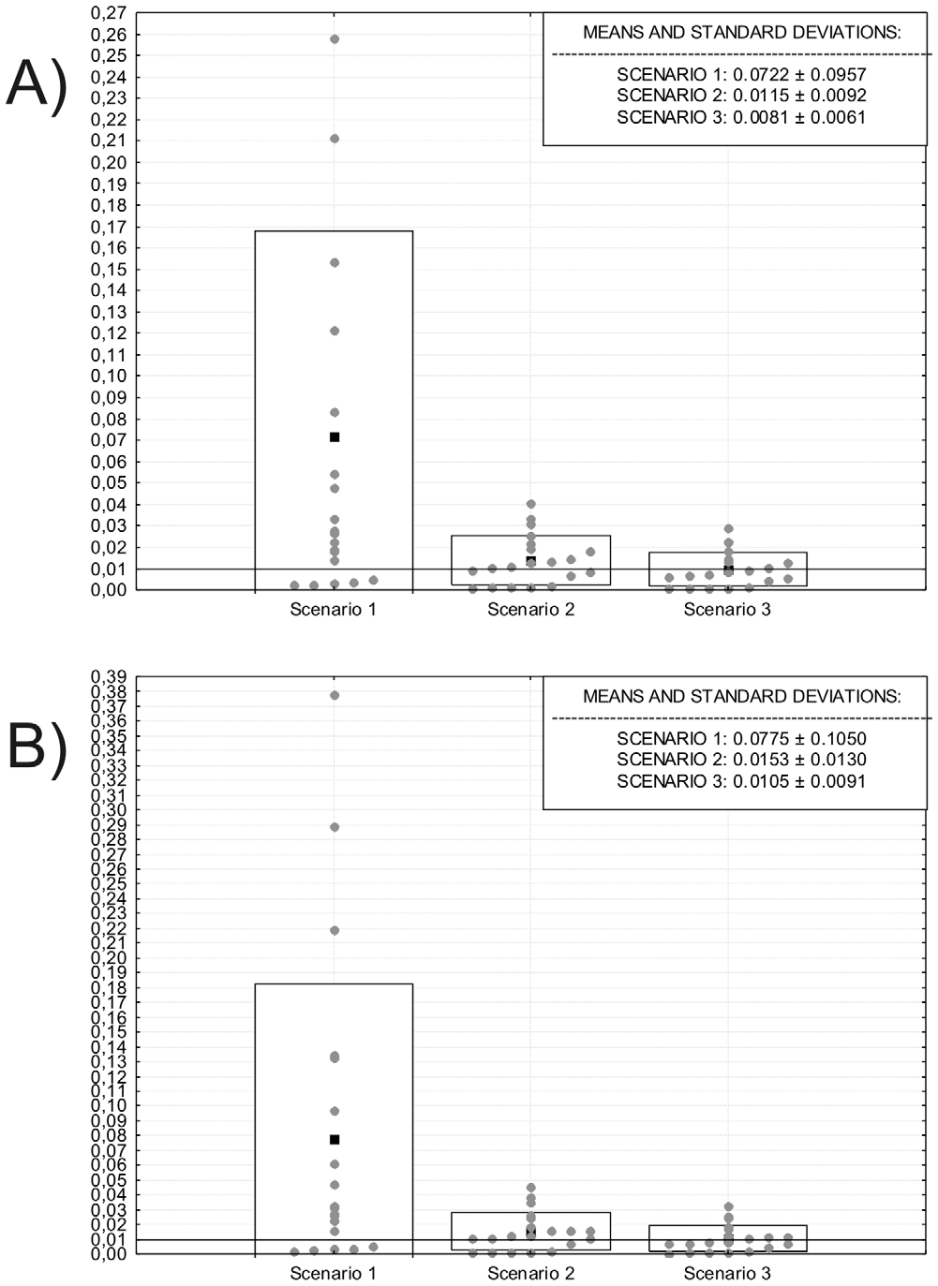
Boxplot of the pairwise mean rates of morphological differentiation (gray dots) calculated between Paleoamerican and Amerindian series. The black squares represent the average of the pairwise mean rates for each scenario and the rectangle represents the confidence limit defined by one time the standard deviation of the mean rates. The black horizontal line shows the upper limit of the neutral expectation range (0.01). A) Results for the 24 variables set. B) Results for the 19 variables set.

Figure 3 presents the mean rates of morphological differentiation calculated for all possible pairwise comparisons between Paleoamerican and Amerindian series, for the two variable sets. The presented results must be interpreted in relation to the reported expected rate of morphological change for mammals under neutral evolutionary expectations, which ranges from 0.0001 to 0.01 [18]. Accordingly, we show very high rates of morphological differentiation in general: in all scenarios a large part of the pairwise comparisons fall above the upper limit of the neutral expectation (0.01). The highest values in all cases are given by the comparison between Archaic Colombia and Peru, as a result of their high between-group variation. In the first scenario the mean rates range from 0.002 to 0.336 for the 24 variables set and from 0.002 to 0.0378 for the 19 variable set, with an average among all pairwise comparisons of 0.07 (24 variables) and 0.08 (19 variables). Clearly these values refute the idea that late Amerindian morphology can be generated through neutral evolutionary processes from the Paleoamerican one. However, as the divergence time is increased (Scenarios 2 and 3), the mean rates calculated approach the neutral limit of 0.01. Differences between the last two scenarios are too small to allow for any differentiation among them, but both scenarios favor the idea that the last common ancestors between Paleoamericans and Amerindians antecedes the arrival of the first human groups in the New World.

Table 1 presents the Mantel test results for the two variable sets. All correlations between geographic and morphological distances were highly significant. Although Mantel correlation tests considering D^2^ matrices tend to underestimate the permutation p-values [28], the very low p-values associated to our analyses favor the interpretation of significant correlation between the matrices. However, the correlation coefficients varied widely, indicating very different levels of support for each of the three models. As expected, the control model (linear distances) showed the lowest correlation coefficient. Furthermore, the removal of variables not associated with population history strengthened the correlations between geographic and morphological distances considerably, especially for Model 3 (bipartite Asian origin). By far the highest correlation coefficient obtained in both runs was for this model.

**Table 1:** Mantel Correlations between Mahalanobis Squared Distances (D^2^) and each of the geographic distance models tested.

Dispersion Model		D^2^ calculated from 24 variables	D^2^ calculated from 19 variables (without variables associated with climatic adaptation)
Model 1	Linear Geographic Distances (Control)	r = 0.24545r^2^ = 0.06025p = 0.0018	r = 0.22735r^2^ = 0.05169p = 0.0048
Model 2	One migration through Beringia	r = 0.24827r^2^ = 0.06164p = 0.0117	r = 0.25660r^2^ = 0.06584p = 0.0115
Model 3	Two migrations through Beringia	r = 0.41192r^2^ = 0.16968p = 0.0004	r = 0.47900r^2^ = 0.2294p = 0.0001

r – two-way Mantel correlation r.

p – associated probability of r after 10000 permutations.

In order to test if Model 3 presents a better fit to the morphological distances than the other three models, Dow-Cheverud tests [29] were applied. Table 2 presents the results of these tests, comparing Model 3 against the other two models. As can be observed, when all 24 variables are taken into account, the bipartite origin of Native Americans cannot be considered a better prediction of the Mahalanobis distances than the other models (i.e. correlations are not significant, although approaching significance). However, when the same test is applied on the set of variables not correlated to climate adaptation, the bipartite origin is clearly a stronger scenario than the control and one-migration models, now showing significant correlations.

**Table 2:** Results of the Dow-Cheverud test between the bipartite model (Model 3) against the other ones.

Model 3 – Two migrations through Beringia *versus*		Dow-Cheverud results based on 24 variables	Dow-Cheverud results based on 19 variables
Model 1	Linear Geographic Distances (Control)	r = 0.14537p = 0.0840	**r = 0.21975p = 0.0143**
Model 2	One migration through Beringia	r = 0.16969p = 0.0656	**r = 0.23062p = 0.0155**

r – two-way Mantel correlation r.

p – associated probability of r after 10000 permutations.

## Discussion

Our results confirm previous observations of large morphological differences between Paleoamericans and Amerindians. They demonstrate that both chronological and geographical models assuming independent origins for these two populations via Beringia fit these morphological differences considerably better than the alternative models. Under the assumption that morphological differentiation among modern humans during the Final Pleistocene and the Holocene was mainly a result of neutral microevolutionary processes [14]–[17], the observed rates of morphological differentiation favor the idea that Early and Late American samples included in this study shared a last common ancestor outside the New World. At the same time, the geographic bipartite model resulted in a generally better fit to the morphological distances among groups. Removal of measurements under selection did not change the pattern of correlation, and in fact strengthened the bipartite model differentially over the other models. Indeed, differences in the obtained r values were significant when the models were compared using the Dow-Cheverud test, when selection was accounted for. However, the removal of variables did not change the results for the mean rates of morphological differentiation between the populations, as would be expected if diversifying selection were responsible for a disproportional increase in the between-group variance among the series.

Our results do not support the hypothesis that the morphological differences between Early and Late American groups are a result of in-situ neutral evolution. Rather they fit better a two wave dispersal model for the settlement of the New World. Our findings are at odds with the majority of molecular evidence on Native American origins [5]–[7] (although they agree with a recent study of rare mitochondrial haplogroups [8] which also favors two origins for Early Americans associated with distinct crossings from northeast Asia within a short period of time [17–15 kyr BP]) .

This disparity between our results and those of most genetic studies points to a large gap in our understanding of the peopling of the New World. Our findings show that this disparity cannot be easily accommodated through selection to climate and that general secular trends appear as a less probable explanation for the morphological differences between Early and Late Native American groups. We propose that the disparity might derive either from diverging sampling strategies between craniometric (that includes both extinct and extant series) and molecular studies (mainly restricted to extant groups); or from the fact that genetic quantitative traits such as cranial morphology might reflect different microevolutionary events from those affecting autosomic or uniparental DNA markers. The first alternative has been proposed before [3]; however recent efforts in recovering ancestral DNA from early Americans have failed so far in identifying distinct mitochondrial lineages in these samples [30]. The second alternative, on the other hand, has received some support based on the fact that parts of the skull morphology respond differentially to environmental pressures [15], [17], [31]. Unfortunately, these possibilities cannot be satisfactorily evaluated until results derived from molecular and morphological data collected from the same populations (extinct and/or extant) are contrasted directly.

We conclude that the morphological diversity documented through time in the New World is best accounted for by a model postulating two waves of human expansion into the continent originating in East Asia and entering through Beringia. This, however, does not completely exclude the possibility that the observed morphological diversity in America is the result of diachronic trends of differentiation [9], or progressive losses of the original variability present in the mother-population of Native Americans [10], especially if strong diversifying selection acted upon the morphological pattern brought into the continent by its first populations. Future work should focus on Middle Holocene samples in order to further test the bipartite model suggested here.

## Materials and Methods

### Samples included in the analyses

The cranial series involved in this study, together with their sample sizes, chronological ranges and main references are reported in Table 3. Together, they represent the human cranial morphological diversity seen in the Americas, East Asia and Australo-Melanesia. Native American samples were included to represent the Paleoamerican morphology from Early and Middle Holocene (Lagoa Santa, Paleo-Mexico, Paleo-Colombia and Archaic-Colombia) and the Amerindian morphology from Late Holocene (Arikara, Santa Cruz, Peru, Base Aérea and Tapera), as described elsewhere [1]–[3], [32]–[33]. The Zhoukoudian Upper Cave skulls (UC-101 and UC-103) were added as representing the ancestral morphology to the series included in this study [23]–[26].

**Table 3:** Series included in the study, population to which they were associated and information on their geographic coordinates, sample size and chronological range.

Series	Population	Latitude	Longitude	Male sample	Female sample	Chronological range	Reference
Mexico Basin	Paleoamerican	19.41°	−99.13°	3	1	∼10 kyr	*1*
Lagoa Santa	Paleoamerican	−19.90°	−43.94°	14	10	11.0–7.5 kyr	*2*
Paleo Colombia	Paleoamerican	4.61°	−74.08°	6	7	11–6.5 kyr	*3*
Archaic Colombia	Paleoamerican	4.61°	−74.08°	10	18	5–3 kyr	*3*
Zhoukoudian Upper Cave	Upper Pleistocene Asian	39.90°	116.38°	1	1	∼30.0–11 kyr	*39*
Base Aérea	Amerindian	−27.59°	−48.53°	12	9	∼1.0 kyr	*2*
Tapera	Amerindian	−27.59°	−48.53°	26	25	∼1.0 kyr	*2*
Arikara	Amerindian	44.36°	−100.35°	42	27	Sub-recent	*32–33*
Santa Cruz	Amerindian	34.00°	−119.75°	51	51	Sub-recent	*32–33*
Peru	Amerindian	−12.10°	−77.05°	55	55	Sub-recent	*32–33*
Buriat	East Asian	52.40°	106.20°	55	54	Sub-recent	*32–33*
Ainu	East Asian	43.05°	141.34°	48	38	Sub-recent	*32–33*
North Japan	East Asian	43.43°	142.85°	55	32	Sub-recent	*32–33*
South Japan	East Asian	33.30°	131.00°	50	41	Sub-recent	*32–33*
Hainan	East Asian	20.04°	110.34°	45	38	Sub-recent	*32–33*
Tolai	Australo-Melanesian	−4.35°	152.27°	56	54	Sub-recent	*32–33*
Australia	Australo-Melanesian	−35.41°	139.11°	52	49	Sub-recent	*32–33*
Tasmania	Australo-Melanesian	−42.85°	147.29°	45	42	Sub-recent	*32–33*

### Morphological Affinity Analysis

Mahalanobis squared distances among sample pairs were calculated (Table S1). This statistic represents the morphological variation between groups as defined by
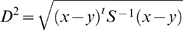



Where x and y are the vectors with the averages of the measurements of each variable for samples X and Y, respectively, and S is the pooled within-group covariance matrix. Consequently, the larger the values of the D^2^ distance, the farther the group centroids are from each other [34]. The D^2^ values were used to compute a minimum spanning tree, a clustering procedure developed for finding the closest “route” for linking a set of points [35], which in turn was superimposed over the geographic coordinates (latitude and longitude) of the series.

### Diachronic morphological differentiation

Under the assumption that inter-species or inter-populations morphological differentiation is a result of neutral evolutionary forces, there is a proportional increase of the between-species variance in relation to the within-species variance for each generation that separates the lineages from its last common ancestor [36]–[37]. From this, Lynch [18] derived a rate of morphological differentiation, given by
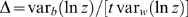



Where var*_b_*(ln *z*) and var*_w_*(ln *z*) are the between-group and the within-group mean squares of an ANOVA for log-transformed measures (z) and *t* is the number of generations separating the lineages from their last common ancestor (i.e. the sum of times down both descendant branches). Lynch [38] reports the expected range of the rate of morphological differentiation under neutral expectation for mammals to fall between 0.01 and 0.0001. Following his methodology [18], here we calculated the mean rate of morphological differentiation across all variables for pairs of populations. The comparisons were made between all Paleoamerican series and all Late American series, changing the number of generations separating them according to three distinct scenarios. In all cases, generation time was assumed to be 20 years, respecting the values used by Lynch [18], which allows for the direct comparison of our data. The age of each series was assumed as the mean of the chronological range presented in Table 3. Howells' series were assumed to be one thousand years old.

The first scenario assumes that Paleoamerican groups are the direct ancestral populations of Amerindians, and as such *t* is defined as the number of generations separating early and late American series. Consequently, this scenario takes into account models for the occupation of the New World that assume only one migration into the continent [9]–[10].

The second and third scenarios, on the other hand, are in accordance with the dual-dispersion model [1]–[3]. The second scenario assumes that Paleoamericans and Amerindians shared they last common ancestor by the time of the first occupation of the continent, around 15 kyr BP [4]–[7], while the third assumes that the last common ancestor between these lineages is represented by the Upper Cave specimens, around 20 kyr BP [39]. Accordingly, *t* for each scenario was defined as the sum of generations down each branch to the date of the last common ancestor assumed.

Since parametric tests of the fitness of the observed rate to the neutral expectation depend largely on the definition of the specific population size (N_e_) [40], which is difficult to estimate for our series, we adopt a qualitative approach here and contrast the range of mean rates of morphological differentiation for the pairwise comparisons against the neutral expectation reported earlier (where modern human samples were also considered and shown to fall within it [18]).

### Geographic range expansion

Still following the assumption that most of the morphological differentiation in modern humans are due to stochastic microevolutionary processes, following isolation by distance or range expansion (resulting from multiple founding effects) patterns of differentiation [10]–[11], [14], [16] with influence of natural selection majorly restricted to extremely cold climates [12], [14], [16], it is expected that the morphological distance between two series should be correlated to the geographic distance separating them if both series departed from a single mother population (or one from another). However, when two populations disperse into the same region distinct from the one where they originally diverged and keep themselves biologically isolated (for example, as in successive colonization events) the geographic distance between them would not be correlated with their biological differentiation, since the real dispersion range of each population is greater than the actual geographic distance between them. Thus, under these evolutionary assumptions, every time the ancestral population of two series is not located in the geographic space between them (either in the same locality of one of them or between them) their biological distance will be poorly correlated to their geographic location. In this situation, a more reasonable model to calculate the distance between these series would be, instead of using linear distances between them, to calculate the distance from the first series to the location of the common ancestral population and from this to the second series.

To test distinct range-expansion scenarios for our data, the Mahalanobis distance matrix was compared to three geographic distance matrices representing distinct models of geographic dispersion using Mantel tests of matrix correlation [41]–[42]. Strictly speaking the correlation coefficient of Mantel's test is simply the coefficient calculated between the off-diagonal elements of each matrix:
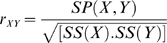



Where
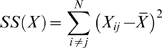


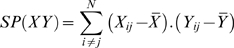



And assuming X and Y to be square distance or similarity matrices with *i* times *j* elements. However, since elements in distance or similarity matrices are by principle not independent, the resulting r has an unknown distribution. The test of the null hypothesis (r  =  0) is done thus through multiple permutations of the rows and corresponding columns of one of the matrices, keeping the other matrix unchanged. In this study p-values were assessed after ten thousand permutations.

To test if any of the geographic models fits better the morphological distances we applied the Dow-Cheverud test [29]. The Dow-Cheverud test permits to test if any of two model (hypothetical) matrices (X_1_ and X_2_) fits better an observed distance matrix (Y) [28]. The test consists of calculating the standardized difference between Matrices X_1_ and X_2_ and correlating it with Matrix Y. If the resulting r is positive and significant (in a new Mantel correlation test) Matrix X_1_ can be assumed as a better predictor of Matrix Y. On the other hand, if the resulting r is negative and significant, then Matrix X_2_ can be seen as a better predictor than X_1_. If r returns a non-significant value, then no matrix can be interpreted as having a better fit to Matrix Y. As a consequence, it was possible to evaluate if the model with the strongest observed correlation with the morphological distances matrix is a better predictor than the other, weaker models.

Although the Dow-Cheverud test has been criticized for being vulnerable when the data shows spatial and/or temporal auto-correlation [43], we chose to apply it here for the following reasons: 1) the geographic models tested must be considered as mutually exclusive, which invalidates its use in the more robust multiple-regression approaches [42]; and 2) Konigsberg [28] demonstrated that in situations like the one considered here, where two arbitrary (and theoretically error-free) model matrices (X_1_ and X_2_) are contrasted against one biological matrix (with an unknown level of random error associated to the measurements), the probability distribution of the Dow-Cheverud test shows no apparent departure from the expected uniform distribution [28: 483].

### The geographic models

In all three dispersion models, the geographic distance assumed for the groups among each population was calculated as the direct distance, in kilometers, between them. This means that the difference between the models relies on how the distance between the groups of different populations is calculated (for example, between Paleoamerican and Amerindian groups).

To contextualize the correlations obtained for the dispersion models, we calculated the first model as a control matrix (Table S2; see Figure 1 for an illustration of the models), which simply represents the direct linear distance between all pairs of series, disregarding such geographic barriers as the Pacific Ocean.

For the two remaining models, distances between series follow only terrestrial routes (using Bangkok, Bering Strait and Panama as “way-points”). Model 2 (Table S3) represents a scenario of local microevolutionary differentiation within the Americas. It predicts that later Amerindians differentiated locally from Paleoamericans, and therefore, following the range expansion model, their morphological differentiation should be proportional to their geographic proximity. It assumes that the differentiation between Early East Asians (Upper Cave, in this case) and Late East Asians occurred prior to the occupation of the New World. Within the Americas distances among series are simple direct linear distances. This matrix represents a settlement model that involved only one major human entrance into the continent, with the morphological variability seen in the Americas through time being the result of in-situ microevolutionary processes.

Model 3 (Table S4) tests distinct origins for Paleoamericans and Amerindians, with both groups representing distinct expansion events into the continent from East Asia through Beringia. In this case, the geographic distance between these groups equals to the distance from the former to Zhoukoudian Upper Cave (passing through Bering and Panama when necessary) plus the distance from Upper Cave to Amerindians (again, through Bering and Panama, when necessary). The remaining distances followed the second model, i.e. no trans-oceanic migrations were allowed.

### Variable sets used in the analyses

The 24 most discriminant cranial variables (Table 4) were selected from an initial database of 40 measurements [32]–[33] by a Back Stepwise Discriminant Analysis (F to Remove/F to enter  =  10/11) [44]. Although uncommon, the stepwise procedure was adopted to minimize the effect that deviations from the population mean as a consequence of small sample sizes have on the Mahalanobis Distance calculations and in the subsequent Mantel correlation tests. Small samples tend to yield poor estimates of the population mean, adding an error to the sample mean which is inversely proportional (although not linearly so) with sample size [45]. For the calculation of the Mahalanobis Distance these random errors bring an additive inflation of the overall distance, since the error associated to each variable will be added in the sum of the final distance value through the difference vector (*x–y*; see formula above). Inflated D^2^ as a product of the additive effect of these random errors will affect especially the results of a Mantel correlation analysis. Due to the nature of matrices representing pairwise comparisons, each sample who gave origin to this matrix influences a large amount of the possible elements of the matrix (2/*N*, while in any linear correlation analysis the weight of any given sample is simply 1/*N*). As a consequence, analysis dependent on such matrices are especially vulnerable to the original error due to the sampling size. In the case of the Mantel correlation analysis, the inflated D^2^ values will result in an increase in its sum of squares [SS(*X*); see above], resulting in a general decrease of the correlation coefficients (exception to this rule will occur when this particular matrix is compared to another matrix were the same elements are inflated, thus increasing significantly their sum of products – SP(*XY*) – that will result in artificially high correlation coefficients). In practical terms, this means that a single small sample can present a higher influence in the correlation coefficient than all the remaining samples included.

**Table 4:** Howells' craniometric variables included in the analyses.

Variables
Glabello-occipital length (GOL)
Nasio-occipital length (NOL)
Basion-bregma height (BBH)
Maximum cranial breadth (XCB)*
Maximum frontal breadth (XFB)*
Nasal height (NLH)*
Bijugal breadth (JUB)
Nasal breadth (NLB)
Bifrontal breadth (FMB)
Biorbital breadth (EKB)
Interorbital breadth (DKB)
Simotic cord (WNB)
Malar length, inferior (IML)
Cheek height (WMH)*
Frontal cord (FRC)*
Frontal subtense (FRS)
Parietal cord (PAC)
Occipital subtense (OCS)
Nasion radius (NAR)
Subspinale radius (SSR)
Zygoorbitale radius (ZOR)
Frontalmalare radius (FMR)
Ectoconchion radius (EKR)
Zygomaxillare radius (ZMR)

*- variables associated with climate adaptation that have been remove in the second run of the analyses.

This effect will be especially marked when the random error of the sample means results in this value appearing slightly outside the range of variation of the remaining sample means, i.e. when the ill-estimated mean presents higher average between-group variance with the other samples than the overall between-group variance for the comparative series. A way to minimize this error is to select the variables to be included in the D^2^ calculation with relatively overall high between-group variation, such as the one applied here through a stepwise discriminant analysis. Although this procedure will overweight the overall differentiation between samples, it will not be affected by the differences resulting from one single sample.

We demonstrate the effect of D^2^ inflation as a consequence of the inclusion of the two Upper Cave skulls as representatives of the ancestral morphology present in East Asia in Supplementary Figures S1 and S2 and Table S5. Together they present the same analyses in the Results, but now based on the complete set of 40 variables. While there is no real change in the pattern of morphological affinities of the series represented in the MST (Figure S1), nor substantial changes in the mean rates of morphological differentiation (Figure S2), we observe an overall decrease in the correlation coefficients (Table S5) when compared to the results section (Table 1). To demonstrate the effect of the two Upper Cave skulls in this analysis, we subsequently calculated the same correlations without the Upper Cave skulls (Table S5). As can be seen, the pattern of correlation coefficients is again very similar to the one presented in our results, corroborating that in the previous analysis the two Upper Cave skulls had more influence in the overall correlation coefficient than the remaining 17 series.

Missing values (9.51% of the measurements) in the prehistoric series were replaced through multiple regression of the variable total mean, using the remaining measurements of each individual as independent variables (but see [46] for a discussion on the topic). Males and females were pooled. For the rate of morphological differentiations, the original data were logged, following [18], to allow for direct comparisons. For the D^2^ based analyses, size effect was corrected by dividing the measurements of each individual by its geometric mean [47]. We accounted for the role of selection by repeating our analyses on a subset of 19 measurements (Table 4) not associated with diversifying selection to climate [13] and thought to mainly reflect population history.

## Supporting Information

Table S1 (0.07 MB DOC)

Table S2 (0.08 MB DOC)

Table S3 (0.07 MB DOC)

Table S4 (0.07 MB DOC)

Table S5 (0.03 MB DOC)

Figure S1 Minimum Spanning Tree of the series calculated from the Mahalanobis squared distances of the complete set of 40 variables and plotted over their geographic coordinates. The lines represent the closest path for connecting all samples according to the morphological distances between them. The presented tree topology is very similar to the one presented for the selected 24 variables, were Early Americans are closely relates to Upper Cave and Autralo-Melanesians, and no direct connection is seen between early and late American series.
(5.65 MB TIF)

Figure S2 Boxplot of the pairwise mean rates of morphological differentiation (gray dots) calculated for the complete set of 40 variables. The black squares represent the average of the pairwise mean rates for each scenario and the rectangle represents the confidence limit defined by one time the standard deviation of the mean rates. The black horizontal line shows the upper limit of the neutral expectation range (0.01). Again, the results observed here are very similar to the ones presented in the results section.
(2.85 MB TIF)
