# Supplementary material for: Testing Evolutionary and Dispersion Scenarios for the Settlement of the New World
Source: PLoS One. 2010 Jun 14;5(6):e11105. doi: 10.1371/journal.pone.0011105 (PMC2885431; doi:10.1371/journal.pone.0011105)
Supplement: Table S1 — (0.07 MB DOC) [file pone.0011105.s001.doc]

Table S1 – Mahalanobis distances between series centroids for the set of 24 selected variables.

|  | ARCHAIC COLOMBIA | UPPER CAVE | PALEO MEXICO | PALEO COLOMBIA | LAGOA SANTA | BASE AEREA | TAPERA | AUSTRALIA | TASMANIA | TOLAI | ARIKARA | SANTA CRUZ | PERU | NORTH JAPAN | SOUTH JAPAN | HAINAN | AINU | BURIAT |
| --- | --- | --- | --- | --- | --- | --- | --- | --- | --- | --- | --- | --- | --- | --- | --- | --- | --- | --- |
| ARCHAIC COLOMBIA | 0.00 | 30.49 | 30.03 | 5.54 | 11.34 | 48.81 | 55.87 | 25.61 | 32.32 | 19.48 | 38.46 | 36.43 | 34.40 | 29.02 | 27.53 | 32.32 | 35.77 | 67.83 |
| UPPER CAVE | 30.49 | 0.00 | 41.39 | 20.50 | 23.39 | 54.24 | 60.68 | 45.66 | 53.91 | 27.79 | 38.62 | 41.12 | 45.89 | 33.72 | 32.45 | 36.80 | 37.50 | 62.78 |
| PALEO MEXICO | 30.03 | 41.39 | 0.00 | 18.56 | 19.47 | 40.65 | 45.84 | 35.33 | 48.42 | 39.57 | 43.41 | 27.86 | 30.01 | 39.94 | 39.53 | 42.68 | 38.21 | 78.05 |
| PALEO COLOMBIA | 5.54 | 20.50 | 18.56 | 0.00 | 7.15 | 39.73 | 44.87 | 22.99 | 30.95 | 18.64 | 28.45 | 23.14 | 25.29 | 22.37 | 21.53 | 25.32 | 24.76 | 54.10 |
| LAGOA SANTA | 11.34 | 23.39 | 19.47 | 7.15 | 0.00 | 43.48 | 52.98 | 26.26 | 30.88 | 20.08 | 32.44 | 26.61 | 25.35 | 22.77 | 21.83 | 23.83 | 25.43 | 59.03 |
| BASE AEREA | 48.81 | 54.24 | 40.65 | 39.73 | 43.48 | 0.00 | 5.74 | 41.54 | 49.60 | 38.38 | 30.68 | 25.17 | 28.99 | 27.91 | 28.83 | 26.82 | 28.04 | 62.97 |
| TAPERA | 55.87 | 60.68 | 45.84 | 44.87 | 52.98 | 5.74 | 0.00 | 46.16 | 54.10 | 46.15 | 33.33 | 23.95 | 30.20 | 31.27 | 33.53 | 32.01 | 31.72 | 63.27 |
| AUSTRALIA | 25.61 | 45.66 | 35.33 | 22.99 | 26.26 | 41.54 | 46.16 | 0.00 | 10.90 | 13.02 | 33.51 | 26.49 | 33.92 | 31.37 | 35.57 | 37.78 | 22.43 | 69.06 |
| TASMANIA | 32.32 | 53.91 | 48.42 | 30.95 | 30.88 | 49.60 | 54.10 | 10.90 | 0.00 | 8.47 | 27.84 | 23.73 | 30.37 | 26.56 | 31.22 | 31.22 | 24.48 | 61.74 |
| TOLAI | 19.48 | 27.79 | 39.57 | 18.64 | 20.08 | 38.38 | 46.15 | 13.02 | 8.47 | 0.00 | 22.43 | 20.84 | 26.90 | 20.03 | 21.37 | 23.26 | 22.43 | 55.99 |
| ARIKARA | 38.46 | 38.62 | 43.41 | 28.45 | 32.44 | 30.68 | 33.33 | 33.51 | 27.84 | 22.43 | 0.00 | 11.70 | 9.22 | 10.93 | 12.10 | 10.53 | 15.55 | 23.88 |
| SANTA CRUZ | 36.43 | 41.12 | 27.86 | 23.14 | 26.61 | 25.17 | 23.95 | 26.49 | 23.73 | 20.84 | 11.70 | 0.00 | 10.42 | 14.58 | 16.79 | 18.66 | 17.56 | 36.57 |
| PERU | 34.40 | 45.89 | 30.01 | 25.29 | 25.35 | 28.99 | 30.20 | 33.92 | 30.37 | 26.90 | 9.22 | 10.42 | 0.00 | 12.99 | 14.34 | 12.01 | 15.96 | 29.07 |
| NORTH JAPAN | 29.02 | 33.72 | 39.94 | 22.37 | 22.77 | 27.91 | 31.27 | 31.37 | 26.56 | 20.03 | 10.93 | 14.58 | 12.99 | 0.00 | 1.82 | 4.01 | 8.20 | 25.50 |
| SOUTH JAPAN | 27.53 | 32.45 | 39.53 | 21.53 | 21.83 | 28.83 | 33.53 | 35.57 | 31.22 | 21.37 | 12.10 | 16.79 | 14.34 | 1.82 | 0.00 | 3.71 | 8.41 | 30.03 |
| HAINAN | 32.32 | 36.80 | 42.68 | 25.32 | 23.83 | 26.82 | 32.01 | 37.78 | 31.22 | 23.26 | 10.53 | 18.66 | 12.01 | 4.01 | 3.71 | 0.00 | 9.91 | 23.39 |
| AINU | 35.77 | 37.50 | 38.21 | 24.76 | 25.43 | 28.04 | 31.72 | 22.43 | 24.48 | 22.43 | 15.55 | 17.56 | 15.96 | 8.20 | 8.41 | 9.91 | 0.00 | 35.77 |
| BURIAT | 67.83 | 62.78 | 78.05 | 54.10 | 59.03 | 62.97 | 63.27 | 69.06 | 61.74 | 55.99 | 23.88 | 36.57 | 29.07 | 25.50 | 30.03 | 23.39 | 35.77 | 0.00 |
