# Supplementary material for: Testing Evolutionary and Dispersion Scenarios for the Settlement of the New World
Source: PLoS One. 2010 Jun 14;5(6):e11105. doi: 10.1371/journal.pone.0011105 (PMC2885431; doi:10.1371/journal.pone.0011105)
Supplement: Table S2 — (0.08 MB DOC) [file pone.0011105.s002.doc]

Table S2 – Geographic distances in kilometers associated with the first geographic model (control).

|  | ARCHAIC COLOMBIA | UPPER CAVE | PALEO MEXICO | PALEO COLOMBIA | LAGOA SANTA | BASE AEREA | TAPERA | AUSTRALIA | TASMANIA | TOLAI | ARIKARA | SANTA CRUZ | PERU | NORTH JAPAN | SOUTH JAPAN | HAINAN | AINU | BURIAT |
| --- | --- | --- | --- | --- | --- | --- | --- | --- | --- | --- | --- | --- | --- | --- | --- | --- | --- | --- |
| ARCHAIC COLOMBIA | 0 | 14969.66 | 3178.573 | 0 | 4280.442 | 4514.826 | 4514.826 | 15206.45 | 14149.06 | 14896 | 5116.084 | 5734.542 | 1889.168 | 13533.34 | 15059.74 | 17252.99 | 13648.82 | 13692.55 |
| UPPER CAVE | 14969.66 | 0 | 12477.43 | 14969.66 | 17127.03 | 18088.04 | 18088.04 | 8702.617 | 9724.23 | 6142.701 | 9963.138 | 9992.751 | 16674.78 | 2226.522 | 1496.358 | 2284.973 | 2103.29 | 1594.32 |
| PALEO MEXICO | 3178.573 | 12477.43 | 0 | 3178.573 | 7442.441 | 7556.343 | 7556.343 | 14102.06 | 13379.01 | 12132.41 | 2780.021 | 2606.653 | 4262.564 | 10615.85 | 12117 | 14596.86 | 10741.16 | 11676.72 |
| PALEO COLOMBIA | 0 | 14969.66 | 3178.573 | 0 | 4280.442 | 4514.826 | 4514.826 | 15206.45 | 14149.06 | 14896 | 5116.084 | 5734.542 | 1889.168 | 13533.34 | 15059.74 | 17252.99 | 13648.82 | 13692.55 |
| LAGOA SANTA | 4280.442 | 17127.03 | 7442.441 | 4280.442 | 0 | 975.3056 | 975.3056 | 13873.45 | 12959.22 | 16809.27 | 9162.785 | 10014.97 | 3641.136 | 17344.47 | 18465.67 | 17350.97 | 17415.37 | 15598.11 |
| BASE AEREA | 4514.826 | 18088.04 | 7556.343 | 4514.826 | 975.3056 | 0 | 0 | 12979.91 | 12031.57 | 15840.14 | 9586.507 | 10162.94 | 3434.519 | 18001.19 | 19402.29 | 17733.33 | 18102.23 | 16573.36 |
| TAPERA | 4514.826 | 18088.04 | 7556.343 | 4514.826 | 975.3056 | 0 | 0 | 12979.91 | 12031.57 | 15840.14 | 9586.507 | 10162.94 | 3434.519 | 18001.19 | 19402.29 | 17733.33 | 18102.23 | 16573.36 |
| AUSTRALIA | 15206.45 | 8702.617 | 14102.06 | 15206.45 | 13873.45 | 12979.91 | 12979.91 | 0 | 1087.522 | 3713.389 | 14976.73 | 13029.79 | 13522.64 | 8785.536 | 7696.131 | 6879.84 | 8737.972 | 10285.16 |
| TASMANIA | 14149.06 | 9724.23 | 13379.01 | 14149.06 | 12959.22 | 12031.57 | 12031.57 | 1087.522 | 0 | 4314.445 | 14745.34 | 12726.32 | 12438.35 | 9615.857 | 8638.991 | 7959.715 | 9581.794 | 11315.2 |
| TOLAI | 14896 | 6142.701 | 12132.41 | 14896 | 16809.27 | 15840.14 | 15840.14 | 3713.389 | 4314.445 | 0 | 11737.1 | 10104.48 | 14282.46 | 5403.011 | 4753.157 | 5328.579 | 5390.712 | 7657.062 |
| ARIKARA | 5116.084 | 9963.138 | 2780.021 | 5116.084 | 9162.785 | 9586.507 | 9586.507 | 14976.73 | 14745.34 | 11737.1 | 0 | 2024.763 | 6712.91 | 8430.713 | 9951.935 | 12219.23 | 8542.69 | 8970.778 |
| SANTA CRUZ | 5734.542 | 9992.751 | 2606.653 | 5734.542 | 10014.97 | 10162.94 | 10162.94 | 13029.79 | 12726.32 | 10104.48 | 2024.763 | 0 | 6836.986 | 8030.134 | 9518.148 | 12017.41 | 8157.018 | 9436.317 |
| PERU | 1889.168 | 16674.78 | 4262.564 | 1889.168 | 3641.136 | 3434.519 | 3434.519 | 13522.64 | 12438.35 | 14282.46 | 6712.91 | 6836.986 | 0 | 14867.1 | 16338.33 | 18854.22 | 14993.99 | 15543.49 |
| NORTH JAPAN | 13533.34 | 2226.522 | 10615.85 | 13533.34 | 17344.47 | 18001.19 | 18001.19 | 8785.536 | 9615.857 | 5403.011 | 8430.713 | 8030.134 | 14867.1 | 0 | 1527.212 | 3987.28 | 129.5655 | 2874.542 |
| SOUTH JAPAN | 15059.74 | 1496.358 | 12117 | 15059.74 | 18465.67 | 19402.29 | 19402.29 | 7696.131 | 8638.991 | 4753.157 | 9951.935 | 9518.148 | 16338.33 | 1527.212 | 0 | 2522.165 | 1410.926 | 2907.318 |
| HAINAN | 17252.99 | 2284.973 | 14596.86 | 17252.99 | 17350.97 | 17733.33 | 17733.33 | 6879.84 | 7959.715 | 5328.579 | 12219.23 | 12017.41 | 18854.22 | 3987.28 | 2522.165 | 0 | 3860.542 | 3620.459 |
| AINU | 13648.82 | 2103.29 | 10741.16 | 13648.82 | 17415.37 | 18102.23 | 18102.23 | 8737.972 | 9581.794 | 5390.712 | 8542.69 | 8157.018 | 14993.99 | 129.5655 | 1410.926 | 3860.542 | 0 | 2796.137 |
| BURIAT | 13692.55 | 1594.32 | 11676.72 | 13692.55 | 15598.11 | 16573.36 | 16573.36 | 10285.16 | 11315.2 | 7657.062 | 8970.778 | 9436.317 | 15543.49 | 2874.542 | 2907.318 | 3620.459 | 2796.137 | 0 |
