# Supplementary material for: Testing Evolutionary and Dispersion Scenarios for the Settlement of the New World
Source: PLoS One. 2010 Jun 14;5(6):e11105. doi: 10.1371/journal.pone.0011105 (PMC2885431; doi:10.1371/journal.pone.0011105)
Supplement: Table S3 — (0.07 MB DOC) [file pone.0011105.s003.doc]

Table S3 – Geographic distances in kilometers associated with the second geographic model (single migration).

|  | ARCHAIC COLOMBIA | UPPER CAVE | PALEO MEXICO | PALEO COLOMBIA | LAGOA SANTA | BASE AEREA | TAPERA | AUSTRALIA | TASMANIA | TOLAI | ARIKARA | SANTA CRUZ | PERU | NORTH JAPAN | SOUTH JAPAN | HAINAN | AINU | BURIAT |
| --- | --- | --- | --- | --- | --- | --- | --- | --- | --- | --- | --- | --- | --- | --- | --- | --- | --- | --- |
| ARCHAIC COLOMBIA | 0 | 12642.48 | 3184.156 | 0 | 4280.442 | 4514.826 | 4514.826 | 22759.29 | 23841.69 | 22002.66 | 5194.782 | 5734.784 | 1889.168 | 11115.8 | 12610.9 | 14860.19 | 11217.69 | 11948.98 |
| UPPER CAVE | 12642.48 | 0 | 14280.12 | 12642.48 | 16922.77 | 17135.29 | 17135.29 | 10116.85 | 11199.25 | 9360.217 | 10013.56 | 10130.85 | 14232.92 | 2226.522 | 1496.358 | 2284.973 | 2103.29 | 1594.32 |
| PALEO MEXICO | 3184.156 | 14280.12 | 0 | 3184.156 | 7464.447 | 7676.964 | 7676.964 | 22606.79 | 23689.19 | 21850.16 | 2780.021 | 2606.653 | 4774.599 | 10963.3 | 12458.4 | 14707.69 | 11065.19 | 11796.48 |
| PALEO COLOMBIA | 0 | 12642.48 | 3184.156 | 0 | 4280.442 | 4514.826 | 4514.826 | 22759.29 | 23841.69 | 22002.66 | 5194.782 | 5734.784 | 1889.168 | 11115.8 | 12610.9 | 14860.19 | 11217.69 | 11948.98 |
| LAGOA SANTA | 4280.442 | 16922.77 | 7464.447 | 4280.442 | 0 | 975.3056 | 975.3056 | 27039.58 | 28121.98 | 26282.95 | 9475.073 | 10015.07 | 3641.136 | 15396.09 | 16891.19 | 19140.48 | 15497.98 | 16229.27 |
| BASE AEREA | 4514.826 | 17135.29 | 7676.964 | 4514.826 | 975.3056 | 0 | 0 | 27252.1 | 28334.5 | 26495.46 | 9687.59 | 10227.59 | 3434.519 | 15608.61 | 17103.7 | 19353 | 15710.5 | 16441.79 |
| TAPERA | 4514.826 | 17135.29 | 7676.964 | 4514.826 | 975.3056 | 0 | 0 | 27252.1 | 28334.5 | 26495.46 | 9687.59 | 10227.59 | 3434.519 | 15608.61 | 17103.7 | 19353 | 15710.5 | 16441.79 |
| AUSTRALIA | 22759.29 | 10116.85 | 22606.79 | 22759.29 | 27039.58 | 27252.1 | 27252.1 | 0 | 1087.522 | 3713.389 | 20247.67 | 26802.08 | 24349.73 | 12025.55 | 10590.53 | 8076.085 | 11897.15 | 11151.41 |
| TASMANIA | 23841.69 | 11199.25 | 23689.19 | 23841.69 | 28121.98 | 28334.5 | 28334.5 | 1087.522 | 0 | 4314.445 | 21330.07 | 27884.48 | 25432.13 | 13107.95 | 11672.93 | 9158.485 | 12979.55 | 12233.81 |
| TOLAI | 22002.66 | 9360.217 | 21850.16 | 22002.66 | 26282.95 | 26495.46 | 26495.46 | 3713.389 | 4314.445 | 0 | 19491.03 | 26045.44 | 23593.1 | 11268.91 | 9833.894 | 7319.449 | 11140.51 | 10394.77 |
| ARIKARA | 5194.782 | 10013.56 | 2780.021 | 5194.782 | 9475.073 | 9687.59 | 9687.59 | 20247.67 | 21330.07 | 19491.03 | 0 | 2024.763 | 6785.225 | 8486.879 | 9981.974 | 12231.27 | 8588.771 | 9320.057 |
| SANTA CRUZ | 5734.784 | 10130.85 | 2606.653 | 5734.784 | 10015.07 | 10227.59 | 10227.59 | 26802.08 | 27884.48 | 26045.44 | 2024.763 | 0 | 7325.226 | 8604.178 | 10099.27 | 12348.56 | 8706.07 | 9437.356 |
| PERU | 1889.168 | 14232.92 | 4774.599 | 1889.168 | 3641.136 | 3434.519 | 3434.519 | 24349.73 | 25432.13 | 23593.1 | 6785.225 | 7325.226 | 0 | 12706.24 | 14201.34 | 16450.63 | 12808.14 | 13539.42 |
| NORTH JAPAN | 11115.8 | 2226.522 | 10963.3 | 11115.8 | 15396.09 | 15608.61 | 15608.61 | 12025.55 | 13107.95 | 11268.91 | 8486.879 | 8604.178 | 12706.24 | 0 | 1527.212 | 3987.28 | 129.5655 | 2874.542 |
| SOUTH JAPAN | 12610.9 | 1496.358 | 12458.4 | 12610.9 | 16891.19 | 17103.7 | 17103.7 | 10590.53 | 11672.93 | 9833.894 | 9981.974 | 10099.27 | 14201.34 | 1527.212 | 0 | 2522.165 | 1410.926 | 2907.318 |
| HAINAN | 14860.19 | 2284.973 | 14707.69 | 14860.19 | 19140.48 | 19353 | 19353 | 8076.085 | 9158.485 | 7319.449 | 12231.27 | 12348.56 | 16450.63 | 3987.28 | 2522.165 | 0 | 3860.542 | 3620.459 |
| AINU | 11217.69 | 2103.29 | 11065.19 | 11217.69 | 15497.98 | 15710.5 | 15710.5 | 11897.15 | 12979.55 | 11140.51 | 8588.771 | 8706.07 | 12808.14 | 129.5655 | 1410.926 | 3860.542 | 2701.561 | 3308.714 |
| BURIAT | 11948.98 | 1594.32 | 11796.48 | 11948.98 | 16229.27 | 16441.79 | 16441.79 | 11151.41 | 12233.81 | 10394.77 | 9320.057 | 9437.356 | 13539.42 | 2874.542 | 2907.318 | 3620.459 | 3308.714 | 0 |
