# Supplementary material for: Testing Evolutionary and Dispersion Scenarios for the Settlement of the New World
Source: PLoS One. 2010 Jun 14;5(6):e11105. doi: 10.1371/journal.pone.0011105 (PMC2885431; doi:10.1371/journal.pone.0011105)
Supplement: Table S5 — (0.03 MB DOC) [file pone.0011105.s005.doc]

Table S5 - Mantel Correlations between Mahalanobis Squared Distances (D^2^) and each of the geographic distance models tested for the complete set of variable. Compare the results presented here with the results from Table 1, to quantify the impact of the D^2^ inflation of the two Upper Cave skulls in the correlation coefficients.

| Dispersion Model | | D^2^ calculated from 40 variables (with Upper Cave) | D^2^ calculated from 40 variables (without Upper Cave) |
| --- | --- | --- | --- |
| Model 1 | Linear Geographic Distances (Control) | r = 0.20356  r^2^ =0.04144  p = 0.0020 | r = 0.26824  r^2^ = 0.07195  p = 0.0010 |
| Model 2 | One migration through Beringia | r = 0.14959  r^2^ = 0.02237  p = 0.1120 | r = 0.27126  r^2^ = 0.07358  p = 0.0118 |
| Model 3 | Two migrations through Beringia | r = 0.25947  r^2^ = 0.06732  p = 0.0248 | r = 0.52625  r^2^ = 0.27694  p = 0.0001 |

r – two-way Mantel correlation r.

p – associated probability of r after 10000 permutations.
